# Automated Processing of fNIRS Data—A Visual Guide to the Pitfalls and Consequences

**DOI:** 10.3390/a11050067

**Published:** 2018-05-08

**Authors:** Lia M. Hocke, Ibukunoluwa K. Oni, Chris C. Duszynski, Alex V. Corrigan, Blaise deB. Frederick, Jeff F. Dunn

**Affiliations:** 1 Experimental Imaging Lab, Cumming School of Medicine, Hotchkiss Brain Institute, University of Calgary, Calgary, AB T2N 4Z6, Canada; ibukunoluwa.oni1@ucalgary.ca (I.K.O.); Christopher.duszynsk@ucalgary.ca (C.C.D.); alex.corrigan@ucalgary.ca (A.V.C.); dunnj@ucalgary.ca (J.F.D.); 2 McLean Imaging Center, McLean Hospital, Belmont, MA 02478, USA; BBFrederick@mclean.harvard.edu; 3 Department of Psychiatry, Harvard Medical School, Boston, MA 02115, USA; 4 Alberta Children’s Hospital Research Institute, Calgary, AB T3B 6A8, Canada

**Keywords:** functional Near-Infrared Spectroscopy, pre-processing, post-processing, channel exclusion, motion correction, LF de-noising, GLM, single subject

## Abstract

With the rapid increase in new fNIRS users employing commercial software, there is a concern that many studies are biased by suboptimal processing methods. The purpose of this study is to provide a visual reference showing the effects of different processing methods, to help inform researchers in setting up and evaluating a processing pipeline. We show the significant impact of pre- and post-processing choices and stress again how important it is to combine data from both hemoglobin species in order to make accurate inferences about the activation site.

## 1. Introduction

Since its inception 25 years ago, the field of functional near-infrared spectroscopy (fNIRS) has grown rapidly, with the number of publications doubling every 3.5 years [1]. The number of publications per year is expected to approach 500 by the year 2020 [2]. Advances in technology and hardware have allowed fNIRS researchers to non-invasively probe neurovascular physiology, with increasing resolution and signal quality. However, with the rapid increase in new users employing commercial software, there is a concern that many studies are biased by suboptimal processing methods [3]. Furthermore, an apparent lack of standardized and automated processing and analysis methods makes it difficult to reproduce studies, and may result in the misinterpretation of data, even by experienced fNIRS researchers. In this manuscript, we set out to provide visualizations to describe how small differences in individual processing steps can significantly influence study results.

There are a vast number of signal processing approaches that have been developed to deal with potential confounders of fNIRS data, including various methods for addressing signal quality (channel exclusion, motion correction) and removing physiological noise (filtering, de-noising). With the wide variety of processing strategies, each with its own advantages and disadvantages, it can be difficult to determine the most appropriate algorithm or methodology to implement.

We attempt to address this issue by first evaluating commonly used and widely available preprocessing methods for channel exclusion, motion correction, and filtering. Simultaneously, we evaluate the performance of several de-noising methods and their interaction with a popular statistical model for detecting activation, namely the general linear model (GLM), under varying hemodynamic response conditions. The purpose of this study is to provide a manuscript with visual descriptions of the effects of different processing methods, to help inform researchers with less fNIRS expertise in setting up a processing pipeline.

In the following sections, we will briefly describe some of the potential sources of noise (a more in-depth discussion of this topic can be found elsewhere [4,5]), and outline some of the commonly used methods for addressing signal quality and removing physiological noise.

### 1.1. Channel Exclusion Criteria

Signal quality can be jeopardized by a number of factors including instrument and environment noise, poor coupling of optodes to the head, artifacts introduced by subject motion, and optical interference by coarse or heavily pigmented hair [6]. These sources of noise and artifact can result in reduced signal-to-noise-ratio (SNR), sharp spikes in the data time series, and/or abrupt shifts in baseline intensity, ultimately leading to inaccurate recordings and misinterpretations [7,8].

**Figure 1.**
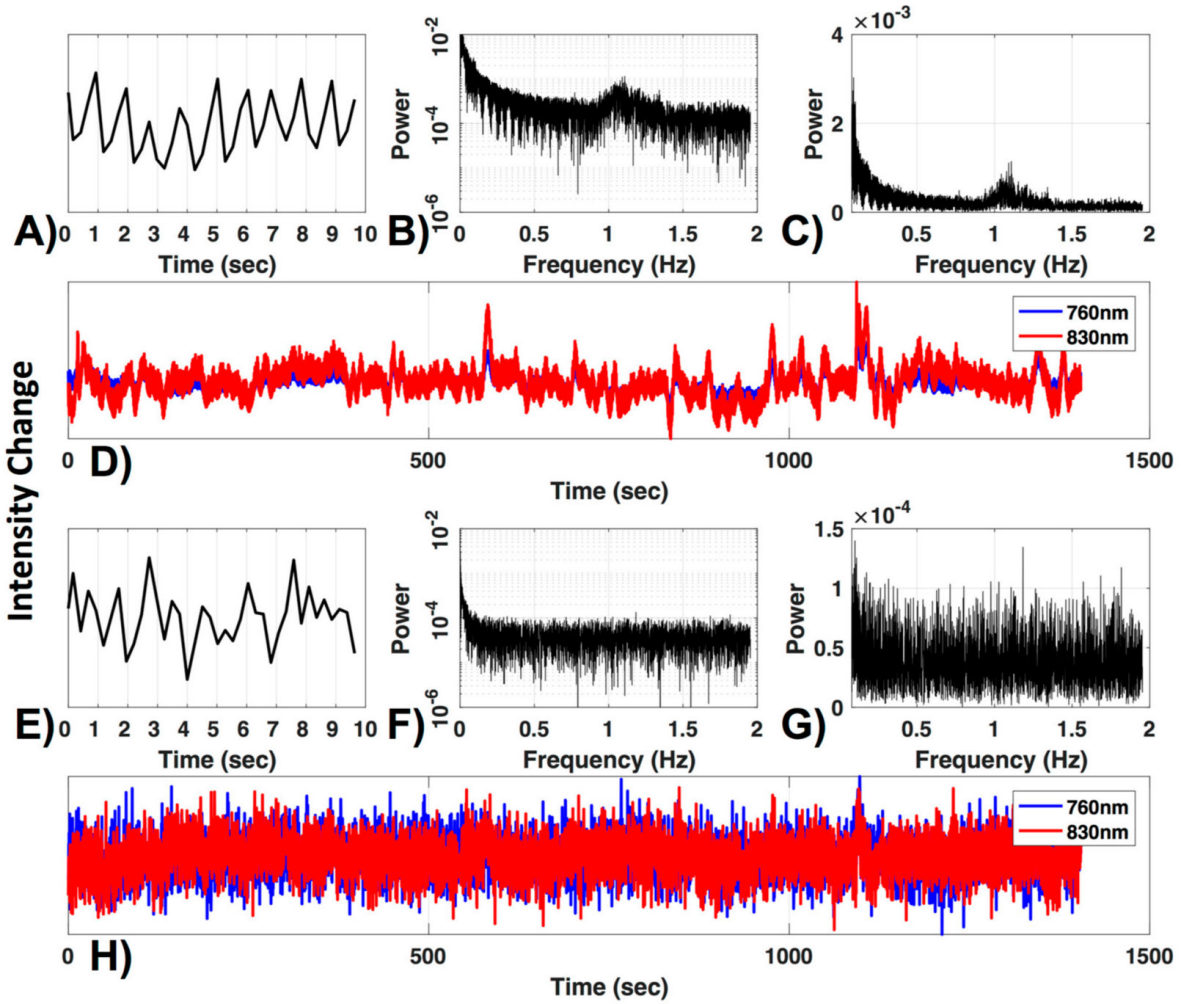
Channel quality. (**A**) A 10-s segment and (**B**) the frequency power spectrum in log and (**C**) normal scale are shown for a 830 nm raw time-course (**D**, red). The typical cardiac waveform in the time domain (**A**) and the typical peak near the 1 Hz cardiac frequency (**B**,**C**) can be observed. Additionally, both wavelengths are plotted (**D**). (**E–H**) show the same figures as (**A–D**) for a channel with low signal to noise ratio.

To ensure adequate signal quality, it is crucial to define a criterion for excluding poor channels that would otherwise contaminate the data. One commonly used criterion is the visual inspection of the ~830–850 nm wavelength optical density signal for presence of cardiac pulsation, in either the time or frequency domain. The rationale being that the presence of a cardiac oscillation, which is robust and consistent, indicates that changes in optical density are coupled with physiological hemodynamic changes. An example of an acceptable channel is shown in Figure 1.

There are, however, several drawbacks to the visual inspection method, i.e., (1) it does not ensure that the ~700 nm wavelength signal is of sufficient quality (this wavelength is weighted to deoxyhemoglobin, and therefore cardiac pulsation is less robust), (2) it is subjective (highly dependent on evaluator), and (3) it is time-consuming, making it inefficient for large datasets. For these reasons, automated methods for channel exclusion are favorable, as long as they perform on par with or better than visual inspection. Several automated methods have been proposed to assess and identify channels for exclusion. The two automated criteria we will investigate are the widely used coefficient of variation (CV) method and the method developed by Pollinini and colleagues [9], named PHOEBE (Placing Headgear Optodes Efficiently before Experimentation), which determines adequate SNR by automatic detection of cardiac pulsation [9].

### 1.2. Motion Correction

It is also crucial to identify and remove artifacts that appear in the signal due to subject motion. Several groups have performed thorough investigations of the available motion correction algorithms [7,10,11]. Results from all three comprehensive comparisons support the use of the wavelet filtering method [12], particularly for dealing with high-frequency spikes, although the spline interpolation method [13] may be better at correcting baseline shifts [10,14].

**Figure 2.**
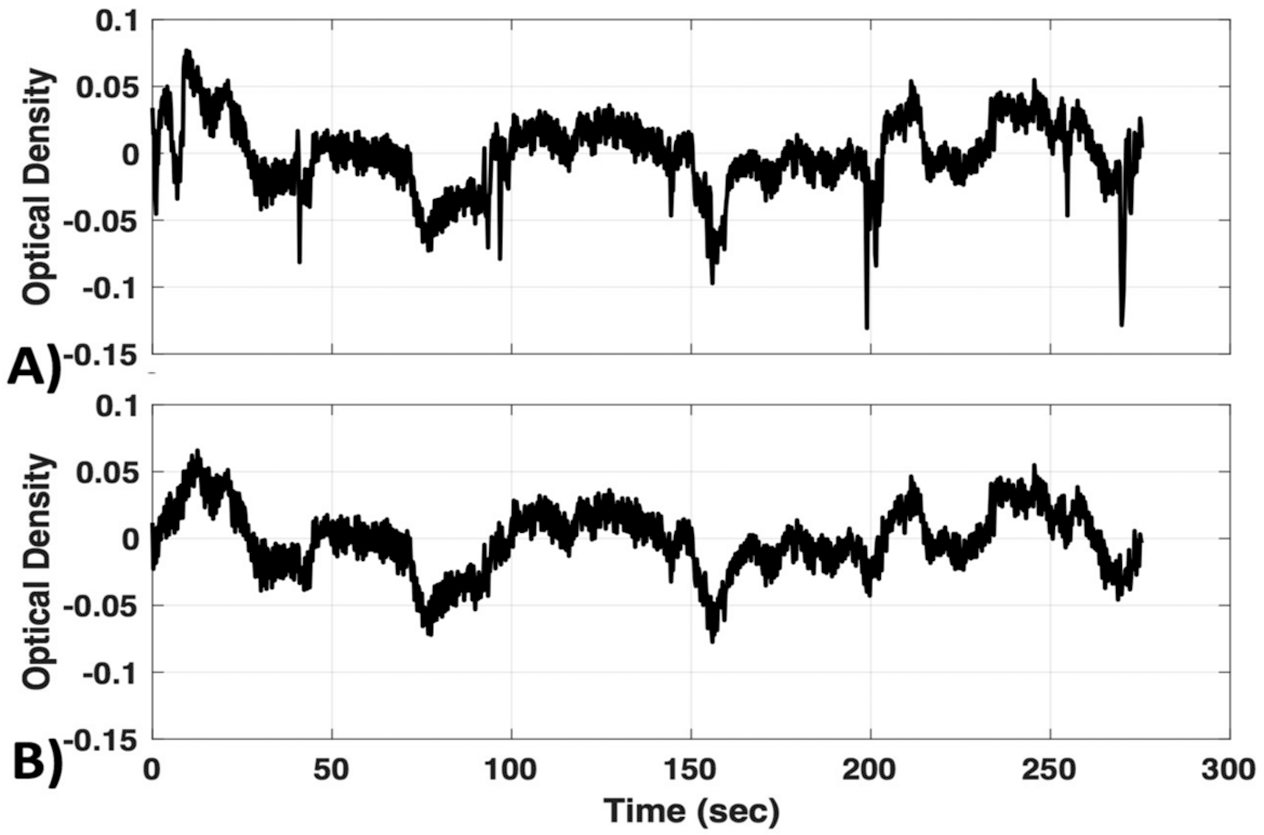
Motion correction. An example of motion correction applied to a signal to successfully remove large spike artifacts. (**A**) Uncorrected; (**B**) motion corrected with the Homer Wavelet motion correction (MC#1) method.

Interestingly, a recently developed method that combines spline interpolation with a smoothing filter (similar in concept to a wavelet filter), appears to improve motion correction of data with both high-frequency spikes and baseline shift components [14]. It is important to note that motion correction algorithms are very powerful tools that have the ability to distort results if used incorrectly. Many of these techniques have numerous adjustable parameters, and the defaults may not be appropriate for all datasets. Even the advanced methods mentioned here may introduce artifacts if called with the wrong parameters. Further, in some cases the efficacy of a motion correction algorithm differs depending on the characteristics of the motion artifact [7,11]. For these reasons a visual inspection of the algorithms’ performance on a subset of data from a given study is recommended instead of assuming the motion correction algorithm you implement with default settings will automatically be correct. In this study we provide a visual comparison of two similar wavelet-based motion correction algorithms (example performance shown in Figure 2) available in the HOMER2 [8] software as well as no motion correction, to highlight the importance of knowing how your motion correction method performs on your data. For a comprehensive analysis of other available motion correction methods, see the studies referenced above [7,10,11].

### 1.3. Filtering and De-Noising for Removal of Systemic Physiology

As discussed by Tachtsidis and Scholkmann [4], a major concern when performing functional NIRS activation studies is the possibility of producing “false positives” and/or “false negatives” by inadvertently measuring physiological components that are non-neuronal in origin, and interpreting the hemodynamic changes as neurovascular coupling [4]. This is particularly troublesome when using “black box” software (software that cannot be accessed in instrument developer software or software that is not understood [4]), which does not account for global or systemic physiology, as is common practice for new fNIRS users [3]. fNIRS recordings are contaminated by both systemic physiology and extracerebral tissue (skin, bone, and cerebrospinal fluid) [15–18]. To avoid producing confounded results, it is helpful to employ measures to reduce the influence of extracerebral tissue and systemic physiology, and increase the weighting of the signal to cerebral tissue.

Bandpass filtering is commonly used to remove high-frequency (≥0.2 Hz; heartbeat, respiratory rate, instrument noise) and very low (<0.01 Hz) frequency information from the signal [8]. Several different filters have been implemented in fNIRS studies for noise removal, with the majority using the Butterworth filter. Other filters used include Chebyshev, elliptic, and zero-phase Fast Fourier Transform (FFT) filters. However, no absolute advantage of one over the other has been observed. For a review on this topic, and on advanced filters that take additional physiological measurements into account, see Naseer and Hong [19]. For the purposes of this study, we offer only a simple visual comparison of two Butterworth filters, and the zero-phase FFT filter.

**Figure 3.**
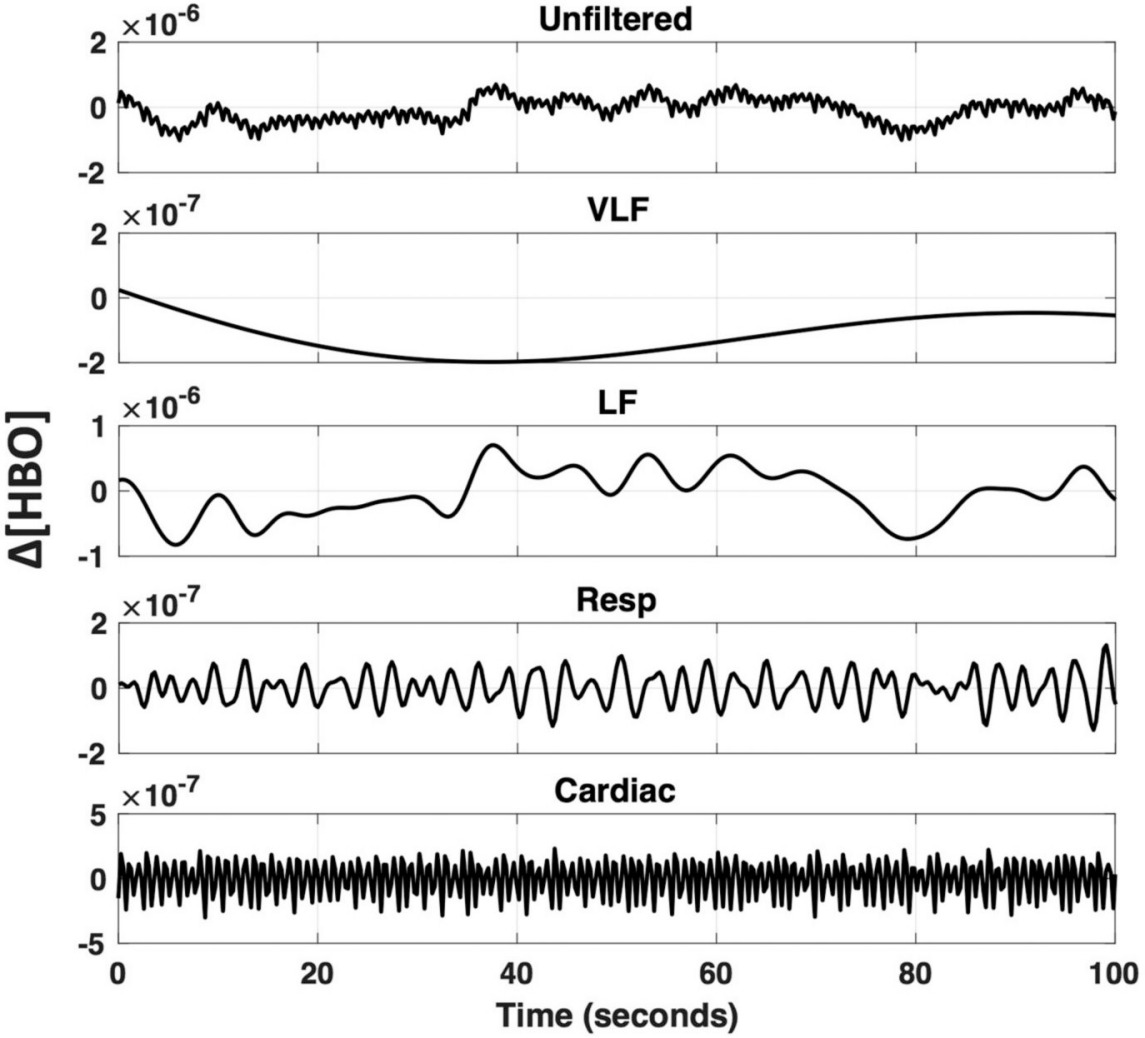
Oscillations underlying fNIRS signals. An fNIRS signal is bandpass filtered to visualize specific frequencies bands that are known to arise from different physiological parameters (VLF—Very Low Frequency < 0.01 Hz; LF—Low Frequency 0.01–0.2 Hz; Respiration 0.2–0.6 Hz; and Cardiac 0.6–2.5 Hz).

Even after bandpass filtering, physiological noise with frequencies that overlap neurovascular coupling frequencies remain in the fNIRS time-series data and can significantly alter results. The low-frequency (LF; 0.01–0.15 Hz) component of the signal is affected by changes in blood pressure, vasoreactivity, CO_2_ concentration, and the autonomic nervous system (ANS), and therefore global or systemic changes can be inadvertently interpreted as neurovascular coupling [4,20]. Furthermore, it has been shown that LF NIRS fluctuations recorded in the periphery are highly correlated with LF fNIRS fluctuations recorded from the surface of the head, as well as LF BOLD fluctuations in the brain, suggesting a major component of the LF hemodynamic signal is of non-neuronal origin [21–26]. A visual example of the different frequency patterns that comprise a typical fNIRS signal is provided in Figure 3, applying a bandpass filter at different frequency bands.

A number of approaches have been developed to increase the sensitivity of fNIRS to neurovascular coupling by removing global physiology. Comprehensive reviews of the aforementioned sources of systemic noise, and the methods available to mitigate them, have been summarized by Scholkmann and colleagues [27] and Tak and colleagues [28]. Three of the more common methods used to separate the neurovascular component from the systemic noise include short source-detector (SSD) regression to remove skin and bone blood flow, and removal of global covariance by either principal component analysis (PCA) or global average (GloAvg). The SSD channel is believed to predominantly measure extracerebral hemodynamics that occur in the skin and bone, and therefore removal of this component increases sensitivity to neurovascular coupling [29,30]. PCA for fNIRS signal processing is data-driven approach that statistically identifies the signal components that explain the greatest amount of spatial covariance (covariance across all spatially distributed channels), and then removes those components from all channels, which also results in a signal that is weighted more towards neurovascular coupling than systemic physiology [31,32]. Removing the global average (GloAvg) signal has also been shown to reduce the influence of systemic physiology; however, it remains a highly debated topic among BOLD and fNIRS researchers due to concerns that it may introduce spurious correlations between spatially distributed signals [33]. More recently, the method used by Erdogan and colleagues that accounts for differences in phase between spatially distributed channels appears to effectively remove global covariance without raising concerns about spuriously increasing correlations [34].

### 1.4. Statistical Evaluation of Task-Evoked Hemodynamics

The general linear model (GLM) is widely used as a statistical method for modeling task-evoked activation in fMRI [35] and fNIRS [36] studies. The GLM allows for the estimation of task activation by explaining the recorded time series as the linear combination of several sources, namely the expected hemodynamic response plus a set of error terms [35]. GLM is often adopted for studying differences between groups or conditions because it is relatively simple to implement using multi-level analysis [28] and between-subject variation is averaged out, leaving significant group differences. However, at the subject level, significant variability in the shape and timing of an individual’s hemodynamic response limits the accuracy of modeling activation with general linear regression [37].

It has been accepted in the fMRI literature that applying derivatives of the hemodynamic basis function can improve GLM estimates by accounting for individual differences in the hemodynamic response [38,39], yet the fNIRS community has only recently adopted the idea. Towards this idea of improving accuracy by capturing individual differences in the hemodynamic response, a recently developed method by Pinti et al. [40] attempts to do just that through the automated detection of various evoked hemodynamic shapes and characteristics. However, further research is warranted to determine this method’s sensitivity and specificity to subject-specific evoked changes.

In recent years, because it is a portable and relatively inexpensive method for non-invasive functional neuroimaging, fNIRS has been increasingly taken up by new researchers interested in measuring brain activity and behavior. Further research and development into novel automated methods for detecting brain activity in real-world situations, such as the one developed by Pinti et al. [40], will accelerate new applications of fNIRS imaging. As the field grows, it is necessary for the fNIRS community to work towards accepted and standardized methodologies. In the meantime, novice fNIRS researchers should remain critically aware of how processing steps can interact to alter their results. This manuscript offers a simple view into the effects of particular preprocessing steps, while evaluating common de-noising methods with respect to their impact on the sensitivity and specificity of detecting hemodynamic activation with general linear modeling, with and without derivatives.

## 2. Methods

### 2.1. Participants

The conjoint health ethics research board of the University of Calgary approved the research. In this study we analyzed 16 participants between the age of nine and 38 (mean = 20 ± 7 years, 12 right-handed, 2 left-handed, and two unknown; five females). Twenty-six participants were originally recruited and measured by newly trained fNIRS operators, who were trained for approximately two weeks to use a NIRX NIRScout. This ensured a reasonable success rate with newly acquainted fNIRS users. Of the 26 participants (mean = 19 ± 7 years, 12 females, two unknown), 10 were excluded due to low SNR, based on a conservative SNR threshold described in Section 2.4.1. Of the 10 participants excluded, two had insufficient SNR in the channel above M1 (primary motor cortex), one had insufficient SNR in both short Source-Detector distance (SSD) channels, and seven had insufficient SNR in M1 and both short distance channels.

### 2.2. Task

Participants were asked to sit quietly in front of a computer screen in a dimly-lit room. The participant was also asked to limit movement. After a rest period, participants performed a finger-tapping task (10 s on 15 s off, for 10 repetitions), in which the right thumb was tapped against the other right fingers sequentially at 1 Hz (timed with the cue “Tap” with the free presentation software OpenSesame [41]). The first participant tapped the thumb against all four fingers at 1 Hz and had only seven repetitions.

### 2.3. NIRS

**Figure 4.**
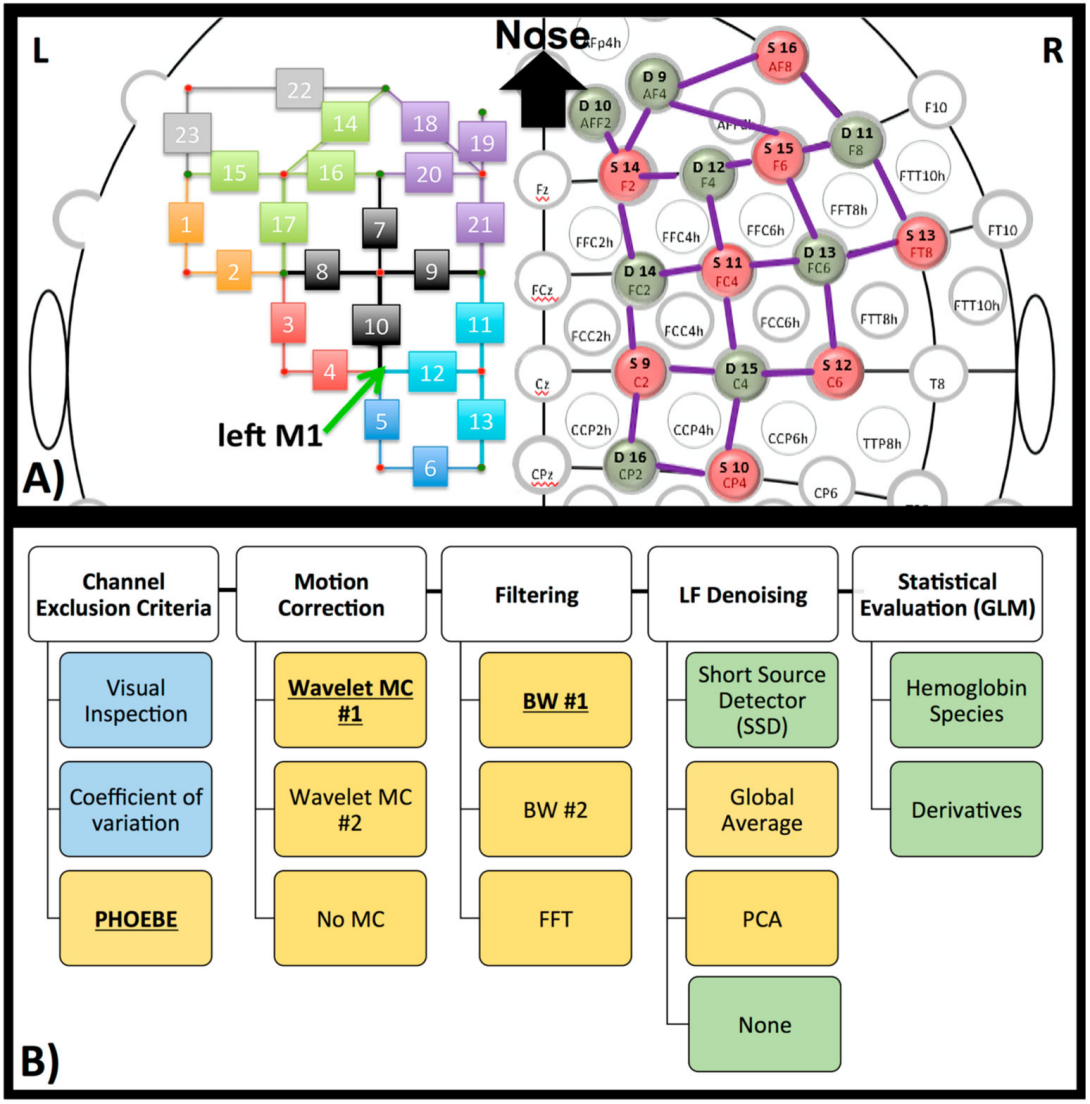
NIRS set-up and processing steps. (**A**) Head-cap configuration, based on the 10–20 system. Sources (red) and detectors (green) are shown on the right hemisphere of the configuration. The symmetrical configuration is presented on the left hemisphere, with channel number rather than source or detector (small green and red circles). Channels are in the same color when the source is identical for these channels. The green arrow on the left hemisphere marks the left primary motor region (M1 coordinate). (**B**) Diagram of processing steps evaluated here. The GLM results later shown in this paper are based on the bolded pre-processing choice. The reasons for these choices are further described in each paragraph in the method and discussion section. Blue represents processing on raw data, yellow on optical density data, and green on concentration data.

FNIRS measurements were continuously collected during all tasks with the NIRScout (NIRX) using the wavelengths 760 and 850 nm at a sampling rate of 3.9062 Hz (the NIRX system is time-multiplexed so the sampling rate is determined by the number of light sources and the illumination pattern used). The 46 channels (16 sources and 16 detectors) were located over the frontal, as well as the motor cortices (Figure 4a). Most channels were “fixed” at 3 cm with NIRX supplied stabilizers (rings, which connect under the probes). However, measurements showed deviations for channel 15 and 16 (2.5 cm) on the left hemisphere as well as the homologous channels on the right hemisphere. In addition, diagonal channels in the frontal cortex, which were not fixed included channel 14 (4 cm) and 18 (4.5 cm) and their symmetrical counterparts on the right hemisphere.

Two SSD channels (Channel 19 and homologous channels on the right hemisphere, source detector distance of 20 mm) were located over the frontal cortex. This is suboptimal—distance is optimally around 8 mm in adult head [42] and should be close to the measurement channel [43]. We chose this distance and location because several hardware configurations do not allow for shorter source-detector distance or SSDs co-localized with each channel. The anatomical locations were measured and marked on the head based on the 10–20 coordinate system. To determine the location of the primary motor cortex (M1), we took 20% of the pre-auricular distance and applied this number from the vertex (Cz) down to the left preauricular point. M1 was therefore located in proximity to detector 7 (homolog to detector 15, Figure 4a), which translates to channels 4, 5, 10 and 12 (Figure 4a, arrow).

### 2.4. Pre-Processing

The pre-processing steps are outlined in Figure 4b. Explanations for the decision of which results to present are outlined in the method and result section. Preprocessing was done, using MATLAB-based software packages (MATLAB 6.1, The MathWorks Inc., Natick, MA, USA, 2000). Raw data was trimmed so only the finger-tapping experiment data was analyzed, to mimic the length of a typical experiment, measuring a specific task. Conversion from raw fNIRS time courses to optical density and then to oxy- (ΔHbO), deoxyhemoglobin (ΔHb) was done with HOMER2 (v2.2) routines (hmrIntensity2OD and hmrOD2Conc, respectively) using the modified Beer-Lambert law [44] with age-dependent differential path length factors (DPF). The typical DPF value of 6, as is suggested in HOMER2, or the often cited DPFs of 6.38 and 7.25 for 850 nm and 760 nm respectively [45], may not necessarily be suitable for participants of all ages. It has been shown previously that the path length of NIR light can change as a function of age [46]. Since we evaluated pre-processing methods on participants within a large age range, we also implemented age as a factor in determining the DPF, as previously shown [47]. The code used to determine age-adjusted DPF is in the Appendix A and was sourced from Scholkmann and Wolf [47]. We should note that this code was validated for frontotemporal regions and may not be accurate for locations as the occipital cortex.

#### 2.4.1. Channel Exclusion Criteria

We first evaluated exclusion criteria of channels on the raw and relative optical density data with three different methods: Visual inspection (VI, as described in the Introduction (Figure 1)), coefficients of variation (CV, e.g., as used in [48]) and PHOEBE [9].

“Coefficients of variation” (CV) is an example of automated evaluation method of the signal in order to exclude channels. CV is given in percentage by CV (%) = 100 × standard deviation (data)/mean (data). “Data” in this case is the raw optical transmission data, because otherwise the mean is close to zero. Channels above a certain threshold (CV > 7.5%, as used in [48]) indicate unphysiological noise and are excluded from further processing.

PHOEBE [9] is a method, which evaluates the similarity of both wavelengths, as well as the power spectra, to determine cardiac power. PHOEBE outputs these measures separately (SCI_status, Pwr_status) as well as a summary of both combined (Complete_status). For this study, PHOEBE was used with the following default hardcoded parameters: Butterworth filter with cut-off frequencies of 0.8 (“fcut_min”) to 1.95 Hz (“fcut_max”); window time (window length in which calculations are done) was set to 3 s (“SCiWindow” = 3); the scalp coupling index (SCI [49]) threshold (“threshold”) was set to ≥0.7 and the power matrix threshold or peak value of power (“spectral_threshold”) was set to ≥0.1. We accepted the channel when 80% of the channel was evaluated as sufficient under the complete_status (MATLAB code for exclusion: Exclude = find (mean (Complete_status) < 0.8)). PHOEBE is used on the optical density data but using it on the raw data did not make a difference (data not shown). We continued with PHOEBE, as it was the most conservative method (see Results).

#### 2.4.2. Motion Correction

For motion correction, we focused on the comparison of two similar wavelet algorithms from the HOMER2 suite. Specifically we chose “hmrMotionCorrectWavelet” [12] (MC#1, also shown in Figure 2; interquartile range (iqr = 1.5))) and “hmrMotionCorrectKurtosisWavelet” (MC#2, with suggested kurtosis (sharpness of the peak in a curve) level of 3.3 [50]) from HOMER2. We continued to use MC#1, which, in comparison to MC#2, did not overcompensate for motion artifacts (see Section 3.3.).

#### 2.4.3. Filter

After motion correction (here the “hmrMotionCorrectWavelet”, MC#1 algorithm), we filtered the data individually with three different filters to test performance of each. We extracted the frequencies between 0.01 Hz and 0.2 Hz, including the relevant frequency of our motor stimulation protocol of 0.04 Hz (1/(10 s + 15 s) = 0.04 Hz) and its first harmonic at 0.08 Hz. The first filter (BW#1) was again from the HOMER2 suite (“hmrBandpassFilt”) a standard third-order Butterworth bandpass filter (MATLAB code “filtfilt”, using separately a lowpass filter and then a highpass filter). For the second filter we used the same third-order Butterworth bandpass filter (BW#2) but did not split the lowpass filter and highpass filter (MATLAB code “filtfilt” with option “bandpass”). As the last filter we used a zero phase FFT bandpass filter (FFT) with lowpass at 0.01 Hz −10% and lowstop at 0.01 Hz, and as well as highpass at 0.2 Hz and highstop at 0.2 Hz +10%. We filtered the data first to the low-frequency (LF) domain to ensure that the following LF de-noising methods target the systemic global interference in the LF range [23,25] and not effects of systemic physiology at irrelevant frequencies (mostly due to cardiac pulsation, respiration [21,51]). We continued with the use of BW#1, as this is a standard bandpass filter and the filters were comparable (see Section 3.3.).

#### 2.4.4. LF De-Noising Methods

After motion correction and filtering, we evaluated the effects of the three LF de-noising methods, including short-source detector (SSD*) subtraction, Principal Component Analysis (PCA), and global average (GloAvg).

There are several studies showing SSD measurements to be advantageous in removing unwanted systemic physiology, compared to PCA and global average. However the recommended short-source-detector separation is at 8.4 mm for the adult head [42], which can be practically challenging or unfeasible with current systems. Therefore, we chose the greatest “short-distance” possible with our hardware, 20 mm, which is still a reasonable approach [52]. Short distance correction was done on the concentration changes of oxy- and deoxyhemoglobin, as is the default in HOMER2. Adaptive filtering (HOMER2 code “enAdaptiveFilteringSS”, adapted from [53,54]) was used for scaling. The input for acceptable short distance separation was increased to 20 mm, and the recommended values for mu and M were 1 × 10^−4^ and 1, respectively. Because 20 mm is suboptimal for this analysis, we are using an asterisk (SSD*) to remind the reader of this fact.

For PC-based global regression we applied an algorithm on the filtered relative optical density data (all channels, excluding short SD measurements). PCA is used in various studies, sometimes as motion artifact removal and sometimes to extract systemic fluctuations such as respiration and cardiac. However, as described before, especially cardiac signals travel with very different speed through the brain than LF fluctuations do [24]. This makes the usage of PCA for LF oscillations, cardiac and respiration together rather disadvantageous. We filtered out the first principal component (“pca” and “glmfit” from MATLAB Statistics Toolbox). The first principal component is the component that has the highest correlation with the global average signal [33]. The resulting time courses were then converted into oxy- and deoxyhemoglobin concentration changes (HOMER2 code “hmrOD2Conc”).

For the global average we used adaptive filtering with delay adjustment, which has been shown to avoid spurious anti-correlations [34]. In short, the goal of this method is to remove the global average from each individual channel by regressing out the average of all significant correlated channels. The steps are detailed in the following: Channel 1 (channel *x*) is cross correlated with all other channels (channel *y*_1_, … , *y*_n_). Channels *y*_1_, … , *y*_n_ are then shifted, based on their optimal delay time in reference to channel 1 (optimal delay time was determined by cross correlation; “xcorr” code in MATLAB). The now lag-adjusted channel is accepted as significantly correlated if its correlation value to channel 1 is r > 0.37 (determined by random correlation simulation) and their optimal delay time is less than ±5 s (it takes 6–7 s for the hemodynamic signal to propagate through the entire brain [26]). The lag-adjusted channels, which were accepted as significantly correlated to channel 1, are then averaged into one “global” signal, which is regressed out of channel 1 using an adaptive filter (adapted filter from HOMER2 code “enAdaptiveFilteringSS”). This process was repeated for all channels. The adaptive filtering was performed on the optical density time-courses, which were then converted into oxy—and deoxyhemoglobin time-courses (homer 2 code “hmrOD2Conc”).

To evaluate meaningful differences between each LF de-noising method, Cohen’s d [55] and the Contrast to Noise Ratio (CNR) as used in Cui et al. [56] were calculated for the region of interest (ROI) (specifically, channels 4, 5, 10 and 12). For Cohen’s d, block averages were created with HOMER2 function (“hmrBlockAvg”) by taking the rest period (“pre”) from 4 s before onset of task to 0 s (end of possible hemodynamic response from the previous task to start of the new task) and the task period (“dur”) from 5 s to 15 s post task-onset (standard 5 s delay plus task duration of 10 s). Cohen’s d was then calculated by: (mean (dur) – mean (pre))/std. (pre). CNR was calculated by: ∣(mean (dur) – mean (pre))∣/sqrt (var (dur) + var (pre)).

### 2.5. Post-Processing

To determine significantly activated channels, oxy- (ΔHbO) and deoxyhemoglobin (ΔHb) concentration changes were evaluated using the general linear model (GLM) provided in the Huppert Toolbox [57], which uses pre-whitening (operation used prior (“pre”) to GLM to make a time series behave statistically like white noise (whitening)) and robust regression. A constant, “mean” term, the regressor itself convolved with the canonical HRF, and its first and second time derivatives (basis = nirs.design.basis.Canonical; basis.incDeriv = true) were used in the GLM analysis. Only the *t*-stats for the main term were used. Using this software, adjustment for multiple comparisons is automatically performed and can be found in the output “SubjStats.q”. For comparison, we also processed the data without the first order derivatives, which does not account for differences in shape and delay of the hemodynamic response, yet is commonly used in studies of activation.

It is still common practice to consider only oxy- or deoxyhemoglobin changes when determining activation in a channel; however, determining activation only when significant changes in both oxy- and deoxyhemoglobin occur has been shown to be more accurate [48] and may improve specificity. Therefore, for this study, we show determination of a significant channel activation under three different conditions: (1) significant increase in oxyhemoglobin signal, (2) significant decrease in deoxyhemoglobin signal, and (3) significant increase of oxyhemoglobin accompanied by significant decrease in deoxyhemoglobin signals.

For the evaluation of all steps and methods we used statistical comparisons, conducted with a paired sample *t*-test, two-sided and corrected for multiple comparison with the Benjamini-Hochberg method [58]. The *p*-values are marked therefore as *p*_c_.

## 3. Results

### 3.1. Channel Exclusion Criteria

**Figure 5.**
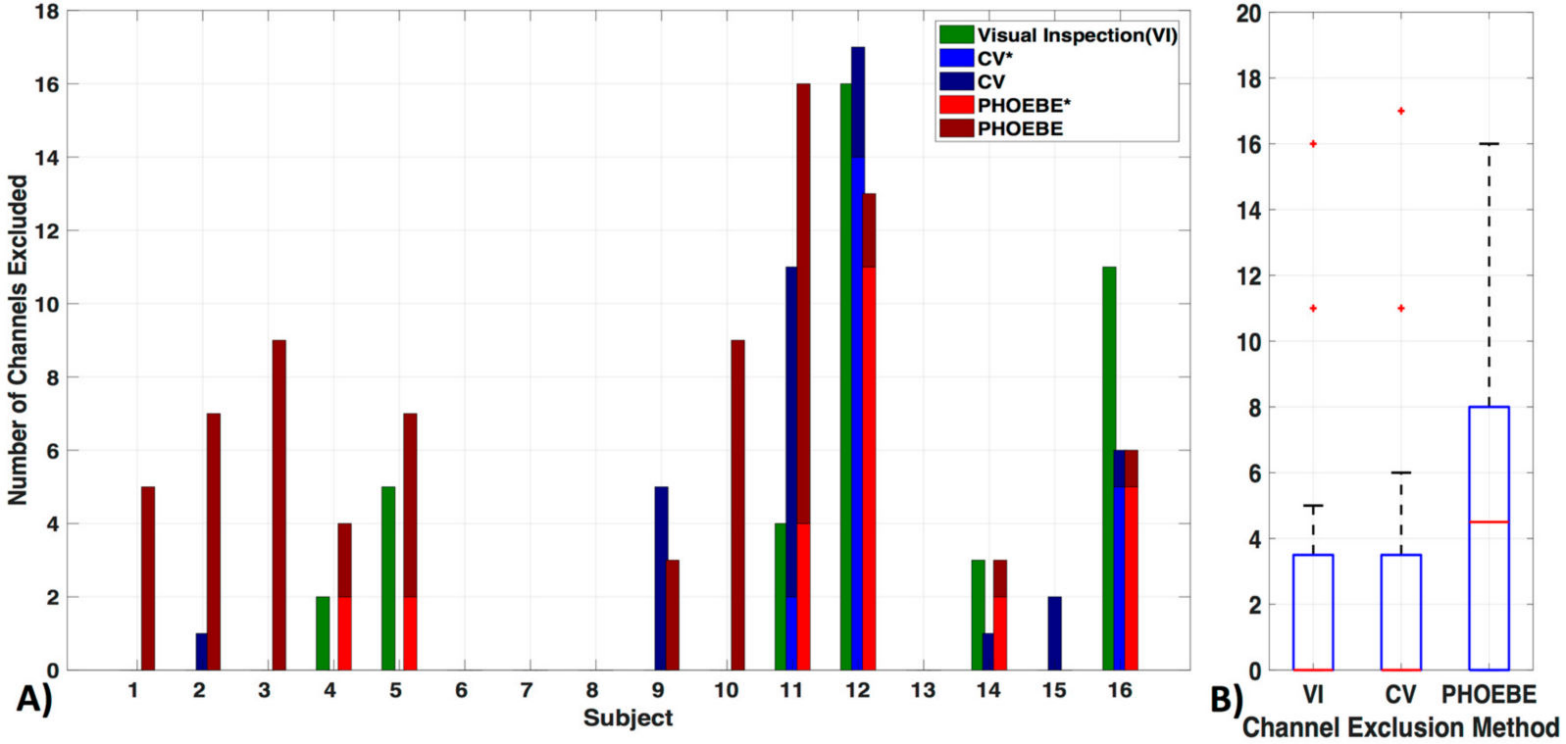
Channel exclusion criteria. Three channel exclusion methods are compared: visual inspection (VI), coefficient of variation (CV), and PHOEBE. (**A**) For each subject, the number of channels excluded are shown in addition to their overlap with visual inspection (‘*’, lighter blue for CV and lighter red for PHOEBE). (**B**) The comparison shows that PHOEBE is the most conservative measurement, excluding more channels than VI or CV.

Exclusion criteria for channels based on the three evaluated methods—visual inspection (VI), coefficient of variation (CV) and PHOEBE were markedly different in each subject (Figure 5a). There was no significant difference between the VI and CV methods (*p*_c_ = 0.8) (Figure 5b). We also did not find significant differences in the number of excluded channels in the VI as well as the CV method in comparison to PHOEBE (*p*_c_ = 0.07), which is likely due to the outliers in the CV and VI methods. Motion artifacts, which were especially high in subject 11 and 12, resulted in higher rejection rates by the CV method, which had otherwise a mean of 2.7 ± 5 channels excluded. In comparison to the VI method excluded 2.6 ± 5 and PHOEBE 5.1 ± 5 channels. In the analyses that follow we used the most conservative measure, PHOEBE.

### 3.2. Motion Correction

**Figure 6.**
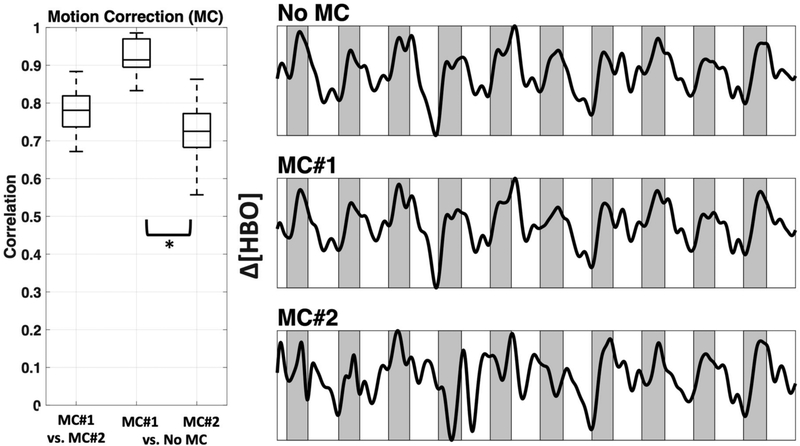
Motion correction. Comparison between two Wavelet MC algorithms, MC#1 and MC#2 (after bandpass filter) for all participants shown as boxplots (**left**). In addition, an example time course for Subject 1 channel 12 is plotted after bandpass filter and PCA (**right**). Shaded sections depict finger tapping.

We used two wavelet motion correction (MC) methods from HOMER2, specifically ‘hmrMotionCorrectWavelet’ (MC#1) and ‘hmrMotionCorrectKurtosisWavelet’ (MC#2). Figure 6 shows the comparison between two motion correction methods, as well as the comparison against no motion correction. The motion correction methods differed significantly in how much they changed the relevant time course (correlation applied after bandpass filter). MC#2 had significantly (*p* < 0.05) lower correlation with the uncorrected data (mean ± std.: *r* = 0.73 ± 0.1) in comparison to MC#1 (*r* = 0.92 ± 0.0). The mean correlation between both methods was *r* = 0.78 ± 0.1. The right side of Figure 6 shows example time courses the channel above M1 (channel 12) in subject #1 after bandpass filter and PCA analysis in three conditions—no motion correction, MC#1, and MC#2. The resulting time courses showed a systematic overcompensation of motion artifacts for MC#2.

### 3.3. Filter

**Figure 7.**
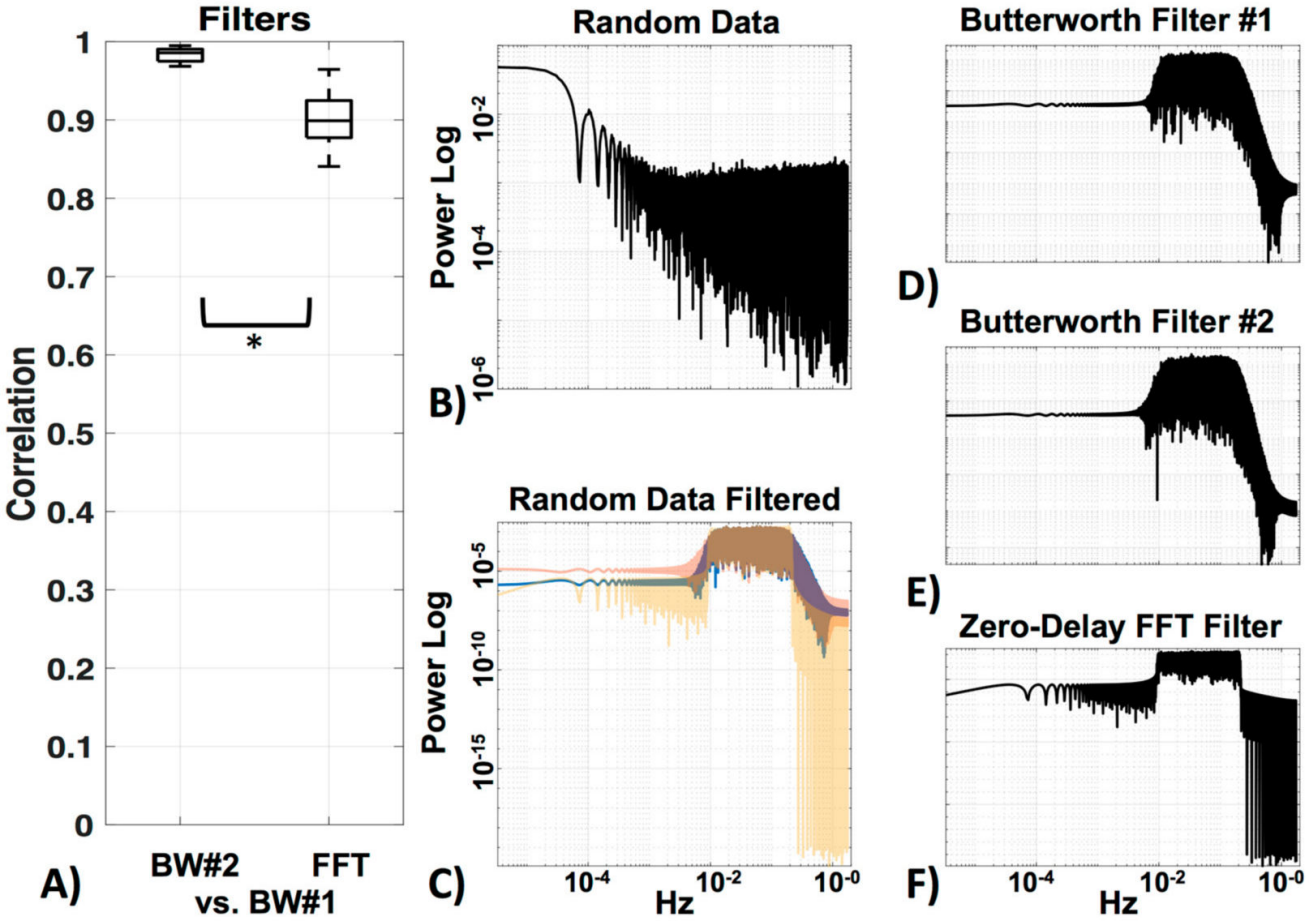
Filters. (**A**) Comparison between the standard HOMER2 Butterworth filter (BW#1) and a Butterworth filter of the same order designed in MATLAB (BW#2), as well as a zero-delay trapezoidal FFT filter (FFT). (**B**) Visualization of power spectra of the random data, (**C**) filtered with all three filters (BW#1 = blue, BW#2 = red, FFT = yellow). Enlargements of all filters in (**C**) can be seen in (**D**) for BW#1, (**E**) for BW#2 and (**F**) for FFT.

We compared three filters—two very similar Butterworth filters, the standard HOMER2 (BW#1), and a Butterworth filter of the same order designed in MATLAB (BW#2), as well as a zero-delay trapezoidal FFT filter (FFT). Figure 7 shows the correlation of BW#2 and FFT with the standard BW#1 (Figure 7a). Random data (MATLAB code “rand”) was generated (Figure 5b) to visualize the power spectrum profile of the filters (Figure 7c–f) on data (the filter profile can also be viewed in MATLAB with “freqz”). The two Butterworth filters were, as expected, very similar (*r* = 0.98 ± 0.0) (see Figure 7c). Their profile showing the long edge in the higher frequencies can be seen in Figure 7d,e. The Zero-Delay FFT Filter shows the typical straight edges at the cut-off frequency (Figure 7c,f). The FFT filter was also highly correlated to BW#1 (*r* = 0.90 ± 0.0), but as expected, significantly less correlated (*p* < 0.05) to BW#1 than BW#2 was to BW#1. In the following analysis, data was filtered with the standard BW#1 to avoid edge effects.

### 3.4. LF De-Noising Methods

**Figure 8.**
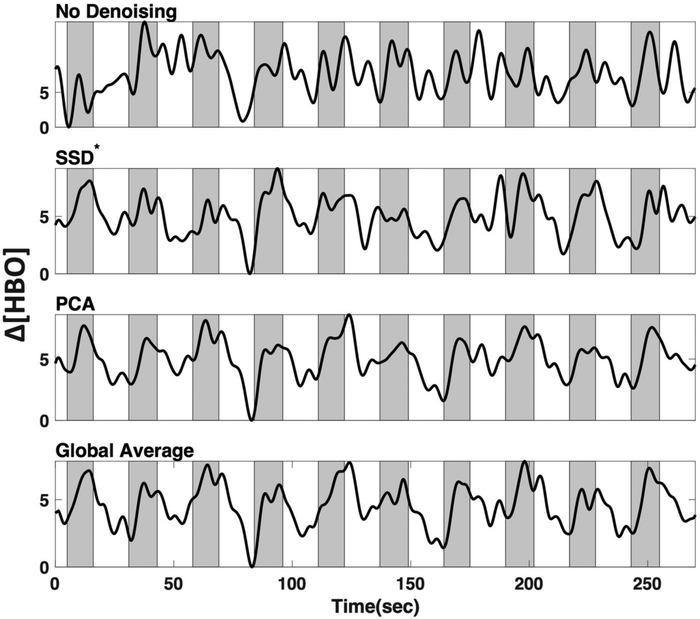
Low-frequency (LF) de-noising methods. Visualization of three commonly used LF de-noising methods, namely short distance adaptive filtering (SSD*, 20 mm source-detector distance), principal component analysis (PCA) and delay-corrected global averaging (GloAvg). Example of changes in oxyhemoglobin from subject #1 in channel 12. Shaded sections depict finger tapping.

We applied three commonly used low frequency (LF) de-noising methods, namely short distance adaptive filtering (SSD*), principal component analysis (PCA) and delay-corrected global averaging (GloAvg). A visualization of an example time course of subject #1 in channel 12 can be seen in Figure 8.

**Figure 9.**
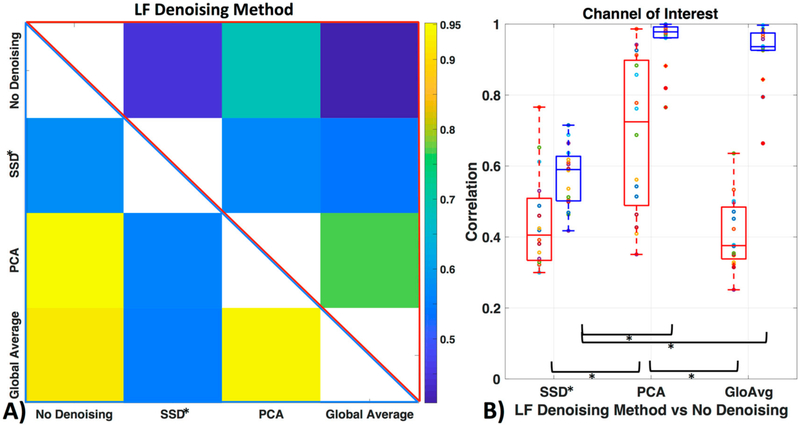
Similarity of LF de-noising methods. (**A**) Correlation between time courses after LF de-noising, with example ROI channel 12. Upper triangle (red frame) depicts mean oxyhemoglobin correlation between the time courses of the different methods and lower triangle (blue frame) shows deoxyhemoglobin. (**B**) Correlation of LF de-noising methods with no de-noising with example ROI channel 12 for oxy- (red) and deoxyhemoglobin (blue).

Figure 9 shows how similar the time courses of the relevant ROI channels are (here channel 12 for all participants). Against no LF de-noising method, we found low to moderate correlations with the oxyhemoglobin time courses (with SSD* *r* = 0.44 ± 0.1, with PCA *r* = 0.70 ± 0.2, with GloAvg *r* = 0.41 ± 0.1) and moderate to high correlation with the deoxyhemoblobin time courses (with SSD* *r* = 0.57 ± 0.1, with PCA *r* = 0.95 ± 0.1, with GloAvg *r* = 0.92 ± 0.1). Between each other, SSD* time courses were moderately correlated with PCA (*r* = 0.56 ± 0.1; *r* = 0.56 ± 0.1 for oxy- and deoxyhemoglobin, respectively) and GloAvg (*r* = 0.54 ± 0.2; *r* = 0.54 ± 0.1) time courses. PCA and GloAvg time courses were highly correlated within oxygenated (*r* = 0.77 ± 0.2) and deoxygenated hemoglobin (*r* = 0.94 ± 0.1).

When comparing similarity of the LF de-noising methods to no de-noising, significant differences with oxygenated hemoglobin were found between the de-noising methods SSD* and PCA (*p*_c_ = 0.0005) and GloAvg and PCA (*p*_c_ = 0.0001) but not between SSD* and GloAvg (*p*_c_ = 0.41). In deoxygenated hemoglobin, differences were found between SSD and PCA (*p*_c_ < 0.05), as well as between SSD* and GloAvg (*p*_c_ < 0.05), but not between PCA and GloAvg (*p*_c_ = 0.17).

**Figure 10.**
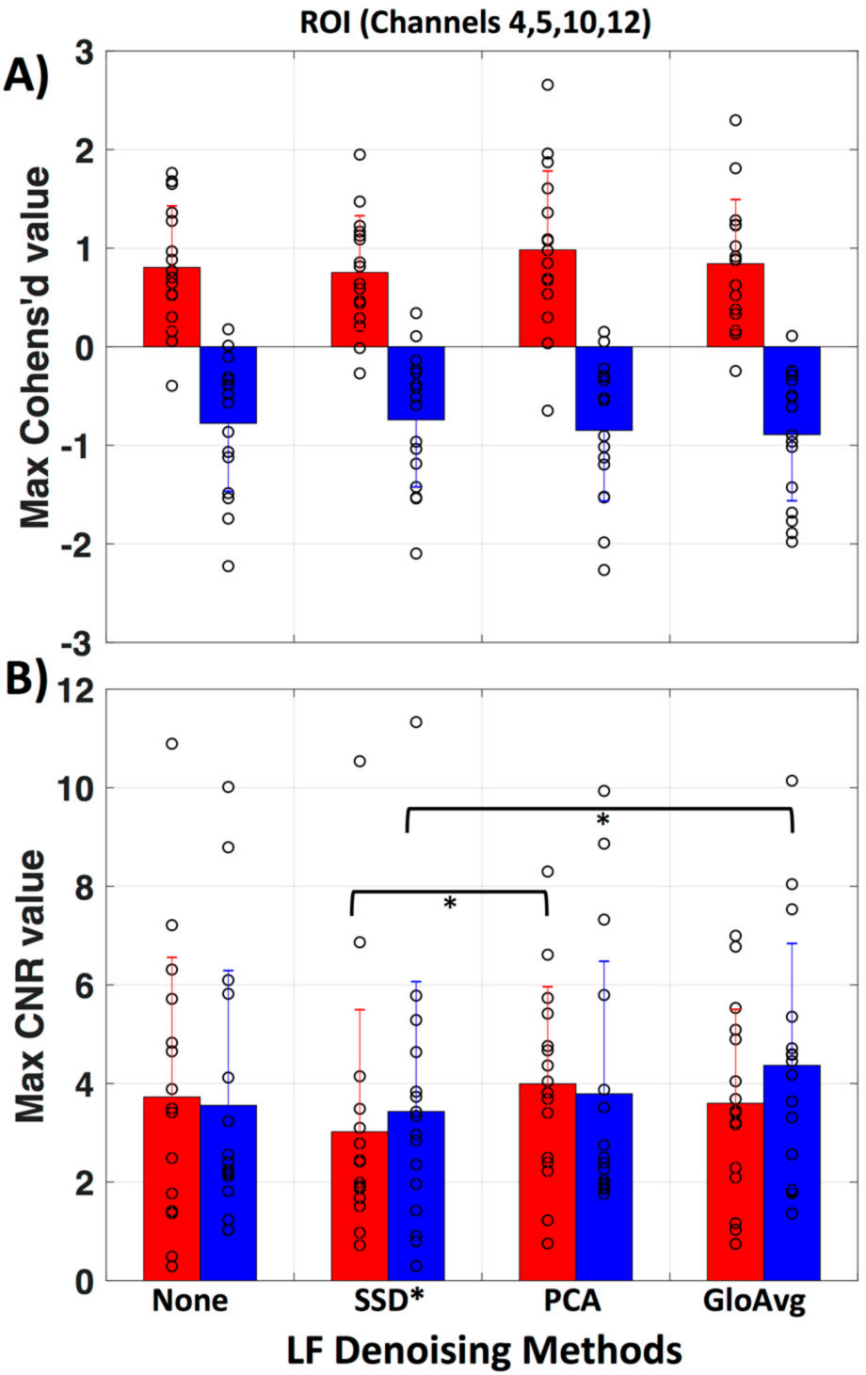
Evaluation of LF methods in regard to Cohen’s d and contrast to noise ratio. (**A**) Mean and standard deviation for maximum Cohen’s d value in the ROI (channels 4, 5, 10 and 12) is presented for no de-noising as well as all three LF de-noising methods. No significant difference was found. (**B**) Mean and standard deviation for maximum contrast to noise ratio (CNR) in the ROI. Red represents oxy- and blue deoxyhemoglobin measures. Each circle represents the maximum value for one subject.

To evaluate if there is a meaningful difference between no de-noising and the LF de-noising methods, we calculated the maximum Cohen’s d (Figure 10a) and contrast to noise ratio (CNR) (Figure 10b) in the region of interest (ROI, channels 4, 5, 10 and 12) for each subject. No significant difference was found for Cohen’s d. Significant lower CNR values were found with the SSD* method in comparison to the PCA method (*p*_c_ = 0.046) with oxyhemoglobin and with the SSD* method in comparison to GloAvg (*p*_c_ = 0.04) with deoxyhemoglobin. Mean maximum Cohen’s d in oxy- and deoxyhemoglobin, respectively was: for no de-noising 0.80 ± 0.6 and −0.78 ± 0.7; for SSD* 0.75 ± 0.6 and −0.74 ± 0.7; for PCA 0.98 ± 0.8 and −0.85 ± 0.7; for AvgGlo 0.84 ± 0.7 and −0.89 ± 0.7. Mean maximum CNR was for no de-noising 3.73 ± 2.8 and 3.56 ± 2.7; for SSD* 3.02 ± 2.5 and 3.43 ± 2.6; for PCA 3.99 ± 2.0 and 3.79 ± 2.7; for GloAvg 3.60 ± 1.9 and 4.4 ± 2.5.

### 3.5. GLM Analysis

**Figure 11.**
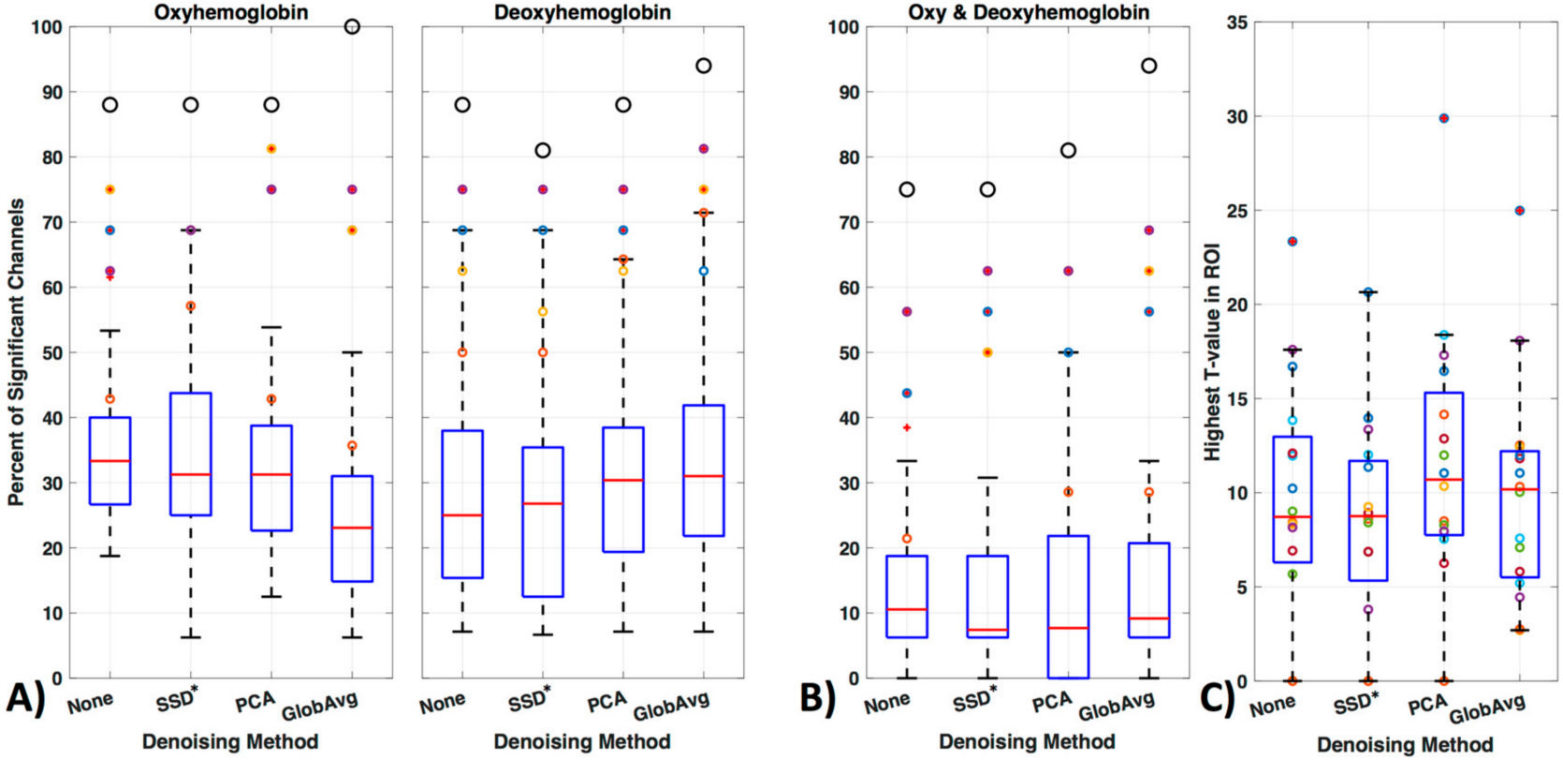
GLM analysis results. (**A**) Results were evaluated with oxy- or deoxyhemoblobin only, (**B**) as well as combined, i.e., when a channel showed a significant increase in oxy- accompanied by a significant decrease in deoxyhemoglobin. The figure shows how many times (in percentage) a channel was regarded as significant over all participants. Outliers are shown as red ‘+’ outside the boxplots 95th percentile and the channels 4, 5, 10 and 12 are shown as yellow, purple, orange and blue colored circles respectively. The larger black circle represents the percentage of times the ROI was activated over all participants, regardless of which or how many channels of the ROI was significantly activated. (**C**) The maximum *t*-values for each subject in the ROI when using the combined hemoglobin condition in (**B**).

We divided our results into three different ways of evaluating significant channels. Specifically, we concentrated on only oxy- or only deoxyhemoblobin (Figure 11a) as well as on regarding a channel only as significant if there is a significant increase in oxy- accompanied by a decrease in deoxyhemoglobin in the same channel (Figure 11b). Figure 11 shows how many times a channel was regarded as significant over all participants in percentage (Figure 11, boxplots). Outliers are shown as red “+” outside the boxplots 95th percentile and the ROI channels 4, 5, 10, and 12 are shown as colored circles (yellow, purple, orange, and blue, respectively); the black larger circle represents the percentage of times the ROI was activated over all participants (regardless of which or how many channels within the ROI were significantly activated).

For oxyhemoglobin alone, 88% of participants had significant activation in the ROI without de-noising, with SSD* and PCA and 100% with GloAvg. For deoxyhemoglobin, 88% of participants had significant activation in the ROI without any LF de-noising and PCA, 81% with SSD* de-noising and 94% with GloAvg. For the combination of significant oxy- and deoxyhemoglobin activation (Figure 11b), 75% of participants had significant activation in the ROI without any LF de-noising and with SSD*, 81% with PCA and 94% of participants had significant activation when using GloAvg.

**Figure 12.**
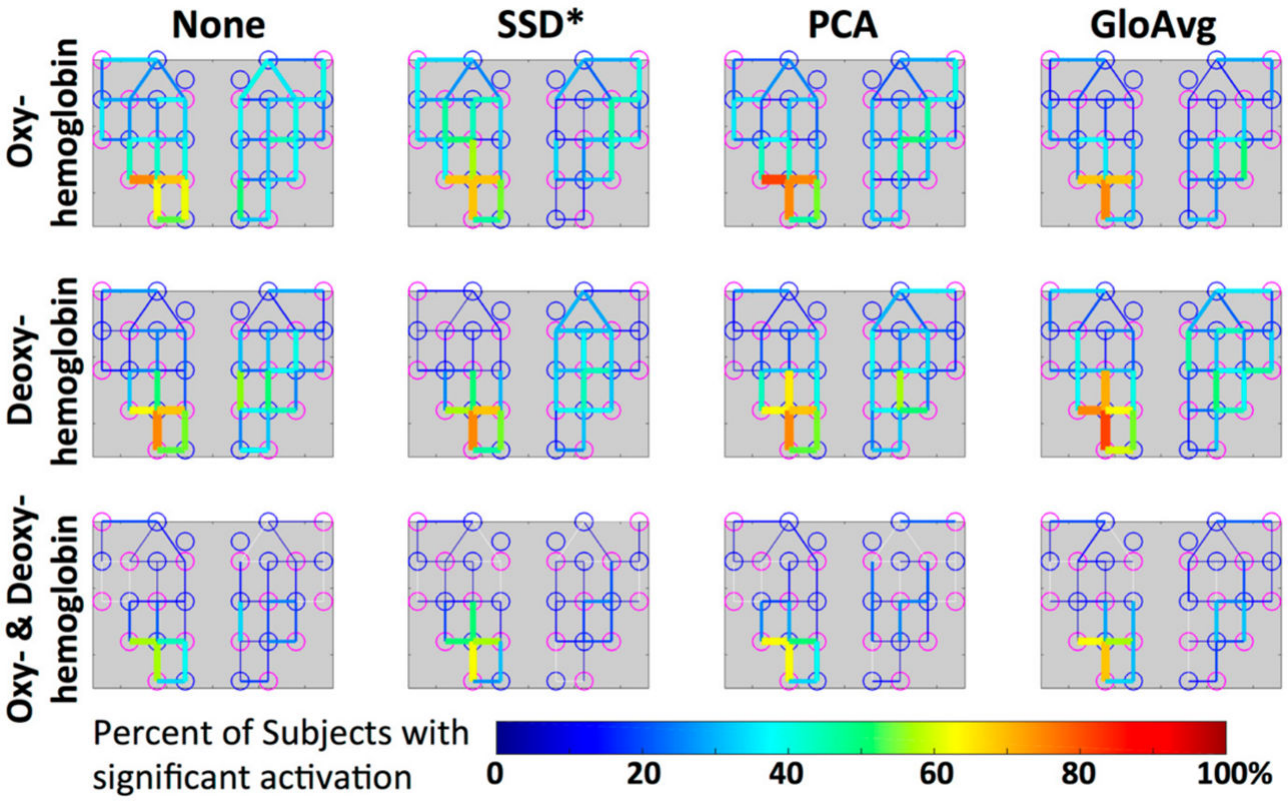
Group level visualization of each channel. Percentage of participants with significant activation in the depicted channel is projected on the simplified channel structure from Figure 3a for all hemoglobin conditions. Lower percentages of participants having a particular channel activated are blue versus higher percentages are red.

The 4 ROI channels and their highest number of activation can be found in Table 1 and Figure 12).

Mean percentage ± standard deviation of significant channels outside of the ROI for oxy-, deoxyhemoblobin and combined were 33 ± 9, 26 ± 13 and 11 ± 10 for None (no LF de-noising); 30 ± 12, 23 ± 13 and 9 ± 8 for SSD*; 30 ± 10; 28 ± 12 10 ± 10 for PCA; and for GloAvg de-noising 22 ± 10, 30 ± 12 and 12 ± 10%, respectively. Significant lower results were found with channels outside the ROI when combining oxy- and deoxyhemoblin instead of oxy- or dexoyhemoglobin alone for all methods (*p*_c_ < 0.000). Differences were also found between oxy- and deoxyhemoblobin alone for all de-noising methods except PCA: None (*p*_c_ = 0.0007), SSD (*p*_c_ = 0.019), PCA (*p*_c_ = 0.38) and GloAvg (*p*_c_ = 0.001).

For the combined hemoglobin condition, the different de-noising methods were significantly higher within the ROI for PCA (*p*_c_ = 0.0005) and GloAvg (*p*_c_ = 0.032) in comparison to no de-noising. No difference was found between: no de-noising vs. SSD* (*p*_c_ = 0.38); SSD* vs. PCA (*p*_c_ = 0.75); SSD* vs. GloAvg (*p*_c_ = 0.93); PCA vs. GloAv g (*p*_c_ = 0.36) (Figure 11, not marked). In addition, the highest *t*-stats did not differ significantly between methods (Figure 11b).

**Figure 13.**
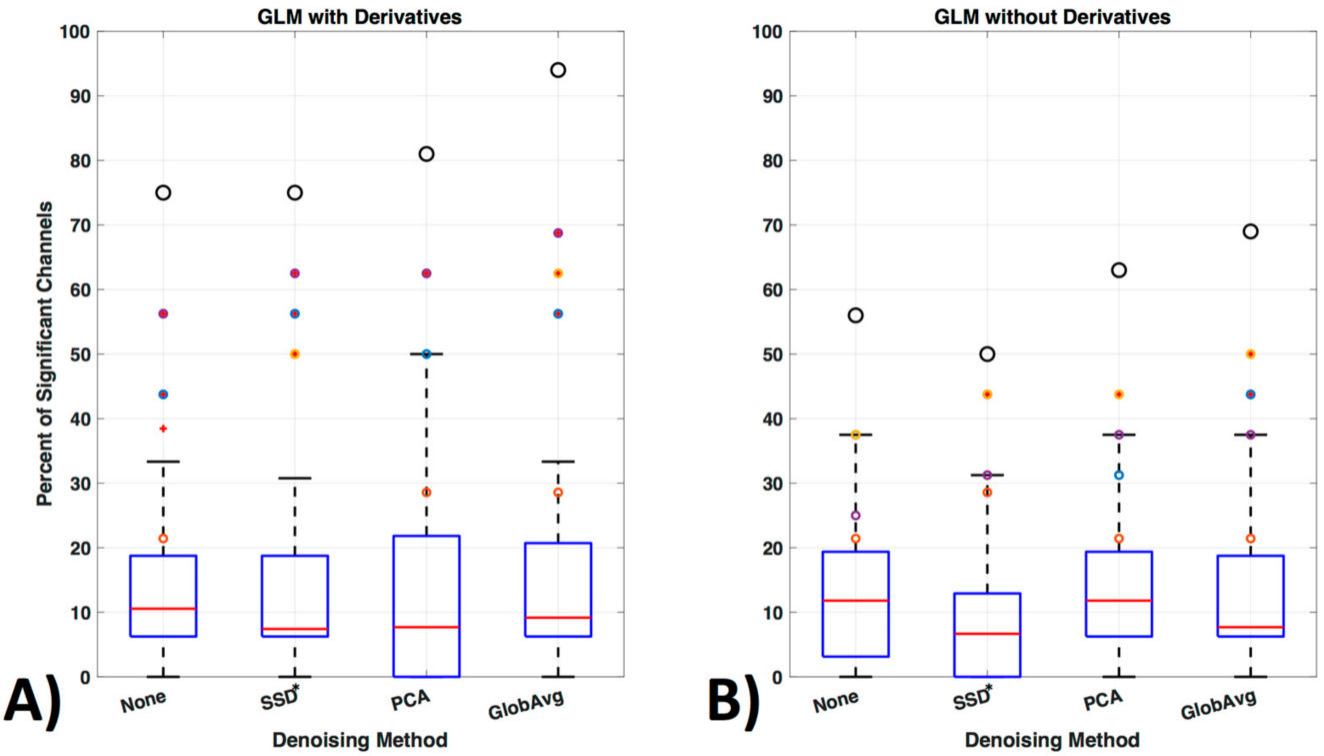
GLM analysis with and without derivatives. (**A**) GLM analysis was originally run with derivatives for the oxy- and deoxyhemoglobin combined condition (see Figure 11b replicated here as comparison) and (**B**) is shown here run without including the first order derivatives to adjust for shape and delay differences in the hemodynamic response function.

We also ran the same GLM analysis without including derivatives (Figure 13). In the oxy- and deoxyhemoglobin combined condition significant lower activity in the ROI over all participants was detected. In addition, within ROI channel activity decreased significantly (*p* = 0.0014) for no LF ne-noising (56%), SSD (50%), PCA (63%) and GloAvg (69%).

## 4. Discussion

The fNIRS field has been highly fortunate to have had a vast choice of free software and continuously updated pre- and post-processing methods. However, with this wide choice of options in processing methods, it is important to realize how these options can influence our results, when comparing studies with different processing options. In addition, we are continuously trying to move towards clinically relevant measures, for which single subject analysis is critical, making small differences, which might be negligible in greater samples, more relevant and problematic. In this study, we evaluated how fNIRS results differ through our choice of several automatic processing methods, especially when we move from group to single subject analysis.

### 4.1. Channel Exclusion

We compared the most commonly used method for evaluating signal quality in a fiber pair, namely visual inspection (VI), with the automatic methods coefficients of variation (CV, e.g., used in [48]), and PHOEBE [9] and found significant differences between these methods (Figure 5). PHOEBE was the most conservative method, excluding channels that may have been accepted with visual inspection. CV was especially sensitive to motion, and otherwise equal to VI, however accepting channels, which would have been excluded with visual inspection. Although that makes PHOEBE recommend itself over CV, careful consideration should be given to the inspection of signal quality, especially as fNIRS configurations often include only a few channels that cover a broad area of the brain, due to the frequent use of 3 cm SD separation. Of course, inclusion of poor channels can lead to misinformation about a brain area, and therefore must be avoided. While we did not find significant differences when using the CV instead of the PHOEBE method to exclude channels (not shown), more differences might be visible with datasets including more noisy channels. On the other hand, loss of good channels, and therefore loss of information in a large area can be highly disadvantageous as well. More problematic is the fact that PHOEBE is specifically designed for the wavelengths used in the NIRX fNIRS system and is unsuitable for use with TechEn data (not shown); however, the developers are working on this issue. The ability for processing methods to work with all systems, in order to retain repeatability between studies [59] is especially important with the increase in different NIRS machines entering the market.

### 4.2. Motion Correction and Filter

Another important consideration for preprocessing is identifying and properly dealing with artifacts that can appear in the signal due to subject motion. With tight coupling of optical fibers, fNIRS is relatively resistant to motion, in comparison to fMRI, however, with increasing use in neonates and natural environments, motion correction is becoming a more important part of the fNIRS pre-processing pipeline. Systematic evaluation of various motion correction algorithms has been performed before [7,10,11] as discussed in the introduction. However here we wanted to show how even two very similar motion correction methods perform differently when used automatically. We found that these two particular methods were quite different (mean correlation between the two wavelet motion corrections was *r* = 0.78, Figure 6). The introduction of larger artifacts caused by one of the motion correction methods with recommended default setting (MC#2) is especially troubling, and can only be detected when visualized. Evaluating which method works on the specific dataset (length of data, specific motion artifacts including spikes as well as DC shifts), instead of trusting that a higher correction (correlation of 0.73 of BW#2 with no correction, versus correlation of 0.92 of BW#1 with no correction) means a better removal of artifacts, is critical and shows that even though there are various motion correction methods, we are not yet at an automatic pre-processing pipeline.

For the bandpass filter, the difference between the bandpass filters we tested was minimal (Figure 7). However, choosing which filter to use can indeed be relevant to data collection and interpretation, as can be seen in the profiles where the FFT filter has very sharp edges and therefore is very precise in the cutoff of the desired frequency in comparison to the Butterworth filter. However, sharp edges can create edge effects, for which it is important to have longer acquisition times before and after the actual task, to avoid these effects in the data itself. Decisions on which processing methods are desired should therefore be made beforehand.

### 4.3. LF De-Noising Methods

The ability to isolate brain activity from low-frequency physiological “noise” has proven to be complicated. In contrast to high-frequency physiological signals, such as respiration (~0.3 Hz) and cardiac pulsations (1 Hz), which can be excluded by bandpass filtering the data (Figures 3 and 7), LF systemic fluctuations cannot be discarded in that way [24,48,51]. LF systemic fluctuations are in the same frequency band as the neuronal signal and travel through the entire body [25]. In addition, the cardiac pulse travels with a very different speed through the brain (closer to a pulse wave) than LF oscillations, stressing the importance of separating removal strategies for these systemic oscillations [24]. Importantly, fNIRS although very similar to fMRI analysis in various domains, cannot necessarily be treated the same as fMRI. Specifically, fNIRS is more prone to systemic fluctuations than MRI is [5,18], due to the path of the photons through all layers of the head and scalp, which ensures substantial contamination of signals with non-neuronal fluctuations. Conversely, removing higher frequency physiological signals such as cardiac and respiratory fluctuations, which is difficult in fMRI, can be achieved through simple spectral filtering in fNIRS.

We tested three common methods that can be used for regression of LF systemic noise, including suboptimal (20 mm) short source-detector (SSD*) recordings, principal component analysis (PCA), and global average (GloAvg) (Example time course, Figure 8). The first two, SSD* and PCA are seldom criticized, yet there is still often the argument against global averaging to produce spurious negative correlations [33]. An advanced de-noising method, which accounts for the time delay between channels, as reported by Erdogan 2016, has shown to remove systemic global variance without inducing spurious correlations in the data, but has not been implemented for fNIRS data.

We filtered the LF noise regressors directly from the data using GLM (PCA) and adaptive filtering (SSD and GloAvg), and evaluated the resulting time courses. We found significant differences in the degree that the LF de-noising methods changed the time course (Figure 9). Specifically, oxyhemoglobin SSD* and GloAvg measures differed greatly from no de-noising, with a correlation of *r* = 0.4. The results of PCA were highly variable (mean correlation of *r* = 0.7). Furthermore, the SSD method was the only method, which greatly affected deoxyhemoglobin measures (correlation with no de-noising of *r* = 0.6 in comparison to *r* = 0.9 for PCA and GloAvg). This is because SSD measures are separately filtered for each wavelength (after dc conversion). We also found significant higher activation across participants in the ROI for PCA and GloAvg in comparison to no LF de-noising in the combined hemoglobin condition (Figure 11b). However, we did not find many differences in how relevant measures, such as Cohen’s d, CNR (Figure 10) or *t*-stats (Figure 11) were affected in the ROI. In addition, which channel was the most activated one across participants varied between LF de-noising methods and hemoglobin species (Table 1). Furthermore, changes in the pre-processing step influenced which method looked more promising as well as which channel was most activated (not shown here).

From the analysis, we can conclude that the PCA and GloAvg de-noising methods, when adjusted for individual delays, perform similarly in fNIRS data (Figure 11). This is in accordance with multiple MRI studies showing that the first PCA component is most similar to the global average [33] and that when the global average is adjusted for delays it performs identically or can outperform PCA [34]. A disadvantage of PCA and GloAvg is that both are influenced by the channels included and the number of channels available. In large datasets these methods work well, but in small datasets the SSD method is highly advantageous. SSD performed similarly or below PCA and GloAvg. However, as described in Section 2.4.4., we did not choose the best possible SSD measure, due to hardware constraints. Various papers have shown increased performance with shorter source-detector separation (e.g., 8.4 mm for the adult head [42], or 6 mm versus 13 mm [60])) and additionally region specific SSD measurements [43] close to the detector [61]. Therefore, our results underestimate the success rate of SSD de-noising. However as long as hardware constraints remain and SSD measures are not directly integrated into the fiber probes, difficulties to use the optimal SSD measure remain. This difficulty includes the sparing, not only of one fiber for the SSD measure and optimally one fiber for each region, but also losing a long distance measurement in the same area with instruments, in which the gain is not adjusted pair-wise (e.g., TechEn).

One variable of many, not addressed in this analysis, is the scaling algorithm. Many exist, including adaptive filtering [53,62], least square minimization (LSM) [63], independent component analysis [64], and Kalman filtering [54], all of which can impact a study’s results. In fact, Gagnon et al. [54] showed improved results with the Kalman filter in comparison to the traditional adaptive filter.

In this study, we evaluate the three aforementioned methods of de-noising, namely SSD*, PCA, and GloAvg, as these are accepted methods that can be easily implemented in any research setting, by users with varying levels of fNIRS experience. One de-noising method not studied here, which has been shown to effectively reduce the physiological noise in fNIRS studies, is the monitoring of systemic physiology (respiration, End-Tidal CO_2_, arterial blood pressure, and ANS responses), with the goal of removing that component from the hemodynamic signal of interest [65–67]. When possible, monitoring systemic physiology can be considered to limit the possibility of false positives and false negatives. The analysis here, however, offers a comparison of de-noising methods that could be used in scenarios that do not allow for the monitoring of systemic physiological parameters.

### 4.4. Hemoglobin

One advantage of fNIRS over techniques such as fMRI is that we can measure the relative change of both oxy-, as well as deoxyhemoglobin at the same time. The balloon model, a model describing the transformation of neuronal activation into a hemodynamic signal, shows a clear pattern of increase in oxy- as well as a slight decrease in deoxyhemoglobin in the region with neuronal activity [68]. Still, various studies use only one or the other hemoglobin change when evaluating the indirect marker of neuronal activity. Whereas oxyhemoglobin is considered to be more closely related to cerebral blood volume and global effects and has a higher signal to noise ratio [69], deoxyhemoglobin is often thought to be more closely linked to regional activity with inherently smaller SNR [70]. We evaluated the sensitivity for oxy- and deoxyhemoglobin as well as the condition in which a significant increase in oxy- has to be accompanied by a significant decrease in deoxyhemoglobin in the same channel.

We found that, a significantly greater number of channels were considered significant in the oxy- or deoxyhemoglobin only condition in comparison to the combined condition (Figure 11). In addition, results for specific channels were less stable in the oxyhemoglobin alone condition between the different LF de-noising methods (Figure 12, Table 1). Especially for no LF de-noising and PCA, the most activated channel across participants changed from channel 4 to channel 5 between oxy- and deoxyhemoglobin respectively, (Table 1). This shows how results can differ when only one hemoglobin species is considered [48], instead of viewing the combined information provided, even when adjusted for LF noise. This is especially true with single subject analysis and with concentration on single channels instead of brain areas [71].

### 4.5. GLM

The fNIRS community has moved from visually inspecting activation and the statistical evaluation of the averaged rest and task period (*t*-test or ANOVA) to the general linear model (GLM). GLM uses the entire time course of fNIRS and its high temporal resolution, which improves statistical power, making GLM the preferred technique [28] in fMRI [72] as well as fNIRS [73]. We chose a commonly used paradigm with repetitive on and off stimuli, which has a high detection power for GLM analysis [74]. However, in our current study, with the usage of standard preprocessing steps such as low-frequency filtering, the GLM has to take into account the facts that (1) hemoglobin time courses are autocorrelated and (2) low-frequency filtering increases this autocorrelation. The result is normally a systematic over-estimation of the error degrees of freedom (lower true number of independent observations) inflating the *t*-values and Type I error [57,75,76]. A comparison of how pre-whitening and robust regression (as used here) can improve the validity of the statistics is described by Huppert et al. [76]. The pre-whitening step is included within the GLM code used here [57]. However, pre-whitening depends highly on the order of how the error term is estimated. Especially since pre-processing steps as low-pass filtering can affect this estimate and therefore the results, it is necessary to continue research on that topic.

In addition it is not considered to be necessary to remove motion with the GLM used here and it is in general not recommended to apply the pre-filtering step (bandpass filter) as a separate step [57], as done in this study. Instead, it is recommended to only filter within the regression model [76], to avoid inflation. However, we did find better results with motion correction and bandpass filter beforehand (not shown). The discussion on what to include in an automated GLM analysis and how to counteract autocorrelation and inflated statistics will likely continue.

Consideration of the shape and timing of the hemodynamic response, not only between but also within participants, remains for all fNIRS statistical evaluations. General practice is to use the default settings of statistical tools such as the gamma function [77,78] in statistical parametric mapping (SPM) or the peak delays of 5 s as hardcoded in the software fOSA [79] for the hemodynamic response function. However, studies have shown that the peak delay and shape may be important variables to study, as they differ between participants, as well as within participants, for each hemoglobin signal, task and brain region [37]. Various papers have modeled the hemodynamic response function, however the importance of the implementation of these models in statistical software packages has only started to be addressed recently (e.g., in the Automatic IDentification of functional Events (AIDE) software using a multitude of shapes and delays [40]). Adding derivatives (temporal and dispersion derivatives), as is standard in fMRI analysis, significantly enhanced detection of activation and increased detection of activation in the ROI channels (Figure 13) and is a step in the right direction.

## 5. Conclusions

We provide guidance for users and a starting point to discuss various pre- and post-processing steps. We show how these parameters can significantly impact the results of task-evoked brain studies with fNIRS. We argue that a consensus is needed to establish an automated processing routine. Furthermore, we showed that default settings can be misleading, and that visualization and additional testing is important when choosing analysis methods. We also introduced a different version of global averaging, which takes the specific delays of the LF noise into account. As a final point, we stress the importance of combining both hemoglobin species in order to improve inferences about where activation occurs. The steps shown in this study should not be considered the ‘best’ possible option since we only tested a certain set of processing methods, but also because the truth of which channels might have been active can’t be determined here. A logical next step is to try to estimate the “true” activation side and compare the methods accordingly. This study is aimed at providing processing guidance and increasing awareness to the importance of validating algorithms. Due to the fact that optimum processing methods have not been established, we continue to support open-source software.

## Figures and Tables

**Table 1.** Activated ROI Channel. Percentage of participants, in which the specific channel within the ROI (4, 5, 10 or 12) was activated, shown for each de-noising method (None, SSD*, PCA, GloAvg) and hemoglobin (oxy- or deoxyhemoblobin alone, or when combined). Highest percentage is shown as bold numbers.

		Oxyhemoglobin				Deoxyhemoglobin			Oxy- & Deoxyhemoglobin			
Channel	None	SSD*	PCA	GloAvg	None	SSD*	PCA	GloAvg	None	SSD*	PCA	GloAvg
**Ch4**	**75**	**69**	**81**	69	63	56	63	75	**56**	50	**63**	63
**Ch5**	63	**69**	75	**75**	**75**	**75**	**75**	**81**	**56**	**63**	**63**	**69**
**Ch10**	43	57	43	36	50	50	64	71	21	50	29	29
**Ch12**	69	**69**	75	69	69	69	69	63	44	56	56	56

## Appendix A

The following MATLAB code (provided by Felix Scholkmann) was used to adjust the DPF for age: function [DPF] = CalcDPF (A, lambda)

% Calculate age-dependent DPF (CalcDPF), Please Insert age for A and wavelength for Lambda

% “General equation for the differential pathlength factor of the

% frontal human head depending on wavelength and age”.

% Please cite Scholkmann, & Wolf, *J Biomed Opt* **2013**, *18*, 105004 for this work

% Parameters

a = 223.3;

b = 0.05624;

c = 0.8493;

d = −5.723E-7;

e = 0.001245;

f = −0.9025;

DPF = a + b.*A.^c + d.*lambda.^3 + e.*lambda.^2 + f.*lambda;

%DPF = sprintf(‘%.2fn’,DPF);
